# Complete Genome Sequence of an *Escherichia coli* O157:H7 Strain Isolated from a Super-Shedder Steer

**DOI:** 10.1128/genomeA.00258-16

**Published:** 2016-04-07

**Authors:** Lin Teng, Amber Ginn, Soojin Jeon, Minyoung Kang, KwangCheol Casey Jeong

**Affiliations:** a Emerging Pathogens Institute, University of Florida, Gainesville, Florida, USA; b Department of Animal Sciences, University of Florida, Gainesville, Florida, USA

## Abstract

We report here the complete genome sequence of *Escherichia coli* O157:H7 strain JEONG-1266 isolated from a super- shedder steer in northwest Florida. Cattle are considered a primary reservoir of *E. coli* O157:H7, and those cattle that excrete this pathogen in their feces at levels ≥10^4^ CFU/g are known as super-shedders.

## GENOME ANNOUNCEMENT

Shiga toxin-producing *Escherichia coli* (STEC) O157:H7 foodborne pathogens are of global concern, as they can cause serious human diseases, such as bloody diarrhea and hemolytic-uremic syndrome (HUS) (1). Cattle are known to be the primary reservoir of STEC O157:H7 isolates. Although the shedding amount of *E. coli* O157:H7 varies from individual to individual and even between cattle of same breed in the same pen (2–4), a subset of cattle termed “super-shedders” are known to shed *E. coli* O157 in their feces at levels of ≥10^4^ CFU/g of feces. Super-shedders were previously identified as the major factor that causes the intra- and interfarm transmission of *E. coli* O157 (5). The phenomenon of super-shedding is believed to be caused by environmental, animal, and bacterial factors (5, 6). In order to elucidate the host-pathogen interactions of super-shedding animals, we report here the complete genome sequence of an STEC O157:H7 strain isolated from a super-shedder steer.

Purified genomic DNA of *E. coli* O157:H7 strain JEONG-1266 was sequenced using the whole-genome PacBio sequencing method (Macrogen, South Korea). By using the PacBio sequencing platform, a total number of 85,386 reads with a mean subread length of 7,660 bases were acquired. *De novo* assembly was performed using the software FALCON, and one contig of chromosomal DNA with a depth of 101× was obtained. The results showed that JEONG-1266 has a chromosome of 5,478,683 bp, containing 5,545 coding sequences (CDSs), 108 tRNAs, and 22 rRNAs. The genome annotation that was deposited in GenBank and the genome sequencing were performed using the National Center for Biotechnology Information (NCBI) rapid annotation pipeline.

Further genome annotation was processed using PATRIC (7), which identified 252 virulence genes, with the majority of genes having >80% similarity to reference genes. Only putative genes having hypothetical protein functions were identified as being unique to JEONG-1266, compared to other extensively researched O157:H7 strains, such as EDL933. An in-depth analysis of the presence/absence of virulence genes in other *E. coli* O157:H7 isolates is under way, as this will provide useful information with regard to host-pathogen interaction and insight into the super-shedding phenomenon. Furthermore, understanding the evolution of this particular strain as it relates to other *E. coli* O157:H7 isolates from low-shedding animals is crucial for the development of mitigation strategies to reduce the occurrence and impact of super-shedding animals. Currently, there are only 157 *E. coli* closed genome sequences available in GenBank. The complete closed genome sequence of JEONG-1266 we present here will facilitate future publications pertaining to the persistence of this *E. coli* O157:H7 strain among super-shedding animals. Lowering *E. coli* O157:H7 from super-shedders is considered an effective strategy to reduce human disease caused by this pathogen.

### Nucleotide sequence accession number.

This complete genome sequence has been deposited at DDBJ/EMBL/GenBank under the accession no. CP014314.
